# Progress in the Remote Sensing Monitoring of the Ecological Environment in Mining Areas

**DOI:** 10.3390/ijerph17061846

**Published:** 2020-03-12

**Authors:** Wen Song, Wei Song, Haihong Gu, Fuping Li

**Affiliations:** 1 Key Laboratory of Land Surface Pattern and Simulation, Institute of Geographic Sciences and Natural Resources Research, Chinese Academy of Sciences, Beijing 100101, China; songwen95@hotmail.com; 2 College of Mining Engineering, North China University of Science and Technology, Tangshan 063210, China; haihonggu1982@hotmail.com (H.G.); tsxyhk@163.com (F.L.); 3 Hebei Key Laboratory of Mining Development and Security Technology, Tangshan 063210, China; 4 Hebei Industrial Technology Institute of Mine Ecological Remediation, Tangshan 063210, China

**Keywords:** mining areas, remote sensing monitoring, ecological environment, review

## Abstract

Based on the results of an extensive literature research, we summarize the research progress of remote sensing monitoring in terms of identifying mining area boundaries and monitoring land use or land cover changes of mining areas. We also analyze the application of remote sensing in monitoring the biodiversity, landscape structure, vegetation change, soil environment, surface runoff conditions, and the atmospheric environment in mining areas and predict the prospects of remote sensing in monitoring the ecological environment in mining areas. Based on the results, the accurate classification of land use or land cover and the accurate extraction of environmental factors are the basis for remote sensing monitoring of the ecological environment in mining areas. In terms of the extraction of ecological factors, vegetation extraction is relatively advanced in contrast to the extraction of animal and microbial data. For the monitoring of environmental conditions of mining areas, sophisticated methods are available to identify pollution levels of vegetation and to accurately monitor soil quality. However, the methods for water and air pollution monitoring in mining areas still need to be improved. These limitations considerably impede the application of remote sensing monitoring in mining areas. The solving of these problems depends on the progress of multi-source remote sensing data and stereoscopic monitoring techniques.

## 1. Introduction

Mining areas are hotspots for the exploration and use of mineral resources and, subsequently, for the deterioration of the natural environment, characterized by conflicts between economic development and environmental protection [1,2]. Since 1973, when NASA started to use earth resource satellites to monitor land use, vegetation coverage, and water accumulation in Appalachian mining areas [3], remote sensing has gradually been developed into an important tool to monitor the ecological environment of mining areas. The development of remote sensing provides convenient conditions for comprehensively, rapidly, and continuously identifying environmental factors and monitoring environmental changes in mining areas. It also provides technical support for decision making by governmental departments.

Mining activities considerably damage the ecological environment, including landscape damage, vegetation damage, agriculture impairment, metal particle deposition, soil erosion, soil pollution, surface runoff, groundwater pollution, air pollution, and waste accumulation [4,5], with serious impacts on the health of local residents and the sustainability of the economic and social development. Generally, when the economy is developing at a medium-high speed, the demand for mineral resources increases rapidly, aggravating the damage to the ecological environment [6]. Using remote sensing instead of time-consuming and laborious traditional methods (e.g., field investigation) to monitor the ecological environment of mining areas can not only improve the management efficiency of mining activities, but also facilitates the evaluation of mining area exploration and ecological restoration [7,8], thus providing technical support for the management of production activities in mining areas.

In mining fields, early remote sensing is mainly applied in mineral exploration [9,10,11], generally by applying surface reflection spectra and images as the identification basis and drawing thematic maps related to mining areas by combining the data with geographic information system (GIS) data. Relevant reviews in this field have also concentrated on this aspect [12,13]. For example, Goetz et al. [14] have summarized the resource exploration situation based on ground survey, aircraft survey, and satellite survey technologies, introduced the remote sensing measurement techniques for rocks, soil, and vegetation, and emphasized the application of hyperspectroscopy in these aspects. In recent years, with the further development and wide application of remote sensing technologies, remote sensing monitoring has been applied more frequently in the monitoring and evaluation of the ecological environment in mining areas. For example, some researchers have dynamically assessed the ecological restoration of mining areas in central Catalonia, Spain, by using multispectral images with a submeter spatial resolution [15]. In another study, which analyzed current land use status in coal mines in southern West Virginia, USA, LiDAR (light detection and ranging) was applied to assess land subsidence [16]. In the MINEO (monitoring the environmental impact of mining activities in Europe using advanced earth observation techniques) project, a European research and technological development project, the environmental impacts of mining activities were investigated, and one of the core tasks was to measure surface radiation in such areas via hyperspectral images, thereby providing conditions for environmental research in mining areas [17].

Although some studies have monitored the ecological environment of mining areas via remote sensing, only a few publications have focused on combining these studies systematically. In this context, based on an extensive literature research, we summarize the research progress in terms of the identification of mining boundaries and land use or land cover changes as well as the monitoring of the ecological environment in mining areas based on remote sensing technology. Our overall aim is to identify the main issues of the current remote sensing technology and to predict the future development direction of this technology.

## 2. Remote Sensing Technology for Ground Feature Types in Mining Areas

The ecological environment of mining areas is complex, with numerous ground feature types, such as vegetation, dumps, water bodies, and tailing ponds, among others. Therefore, the accurate identification of the mining area scope and the precise extraction of various ground features and their changes are the foundation for remote sensing monitoring of the ecological environment in such areas [18]. The use of high spatial resolution remote sensing data, hyperspectral remote sensing data, and topographic data makes the remote sensing monitoring of ground feature types and their changes in mining areas feasible. The basic idea of remote sensing monitoring is the comprehensive use of spatial information, spectral information, elevation information, and angle information. Remote sensing identification of ground feature types mainly focuses on the identification of mining boundaries and land use/cover changes in mining areas.

### 2.1. Identification of Mining Boundaries

Traditionally, the boundaries of mining areas are determined based on the mining area scope specified by the mining license. According to the given coordinates of inflection points, the mining area scope can be formed by connecting these points. However, along with the prospection, exploration, and exhaustion of mineral resources, the specified scope of the mining areas may exceed or be less than the permitted scope. In this sense, the identifying the changes in mining boundaries is particularly important for studying the environmental impacts of mining activities. Identifying the boundaries of mining areas via remote sensing can provide a referenced study area boundary for environmental change assessment in mining areas [19,20]. In this way, the effects of mining activities on the ecological environment inside and outside mining areas can be distinguished. In addition, the accurate identification of mine boundaries could also facilitate the dynamic monitoring of illegal mining activities [21].

Currently, the identification of mining boundaries often adopts relatively mature extraction methods of remote sensing information and combines them with the ground feature environment and ground surface settlement characteristics of mining areas to extract the mining area boundaries (Table 1). According to the unique spectral and textural characteristics of the different ground feature types in mining areas, the fuzzy clustering method is generally adopted to integrate ground feature pixels at a certain distance within the space scope into point clusters, thus extracting boundary change information [20,22]. Notably, this method requires that the extension scope of mining areas is relatively concentrated in terms of spatial distribution. For example, Li et al. [23] have used the fuzzy iterative self-organizing data analysis technique algorithm (ISODATA) clustering method to dynamically identify the boundary of the mining-intensive development zone and extracted the variation range of mining areas from the Landsat (Land Satellite) land use data of Qian’an City in 1987, 1992, and 2001, which were acquired from the interpretation of remote sensing images; their results were consistent with the existing boundary. The mining area reflectivity data set can also be used as the basis for mining boundary identification [24]. By extracting five types of raster data, namely the normalized difference vegetation index (NDVI), the normalized burning ratio index (NBRI), the normalized difference moisture index (NDMI), shortwave infrared data, and red reflectance data, as the training samples, multivariate normal distribution fitting can be performed to find the approximate scope of potential mining areas, however, the boundary has a certain randomness. Here, NDVI data play an important role in boundary identification and in the location of mining areas [25]. In densely vegetated areas, the NDVI data difference based on time series can effectively reflect the land expansion of mining areas based on the original data. The mining area boundary identification method, dominated by the human visual system, is highly precise [26] and uses the snake model to carry out curve evolution for the mining area boundary information extracted from the high-resolution images; its accuracy in mining area boundary identification can exceed 80%. In addition to the above-mentioned identification methods, the combination of high-precision digital elevation model (DEM) data and differential Interferometric synthetic aperture radar (D-InSAR) technology can also effectively extract the mining area scope with severe surface deformation [27,28], and the accuracy of the boundary scope is closely related to the data accuracy and the variation degree of ground types. However, it is difficult to identify the boundaries of mining areas by using only DEM [27,29].

At present, most remote sensing data for mining area boundary identification are derived from the Landsat series of the United States, and the spatial resolution of 30 m facilitates the acquisition of information of large mining areas or mining-intensive areas [22,23,24]. These satellite data can be easily obtained online free of charge, providing convenience for continuous monitoring. However, the extraction of the boundaries of smaller mining areas, or the study of the details of the dynamic changes of the boundaries, requires commercial satellites with higher resolution [26]. Regarding the study of the boundary geodetic patterns of mining areas, the combination of microwave remote sensing with the digital elevation model should be preferred [27,29].

Identifying the changes in mining area boundary based on time series data is generally closely related to the land use changes in such areas. The change of either party can produce linkage reactions, especially when the scope of mining rights is not updated in time. This is also helpful to understand the relationship between boundary identification and ground feature type transformation, facilitating the accurate division of mining areas and the correct evaluation of the ecological environment.

### 2.2. Land Use/Cover Changes in Mining Areas

With changes in mining activities, the land use/cover of mining areas often changes dramatically. Therefore, investigating the land use/cover changes in mining areas is also one important research aspect related to the remote sensing identification of mining area factors. The important land use/cover types in mining areas generally include stopes, subsidence areas, tailing ponds, water areas, and bare land. Remote sensing identification and classification of water areas, bare soil, and slurry tailings discharged after ore washing in mining areas are complex and difficult to perform without prior knowledge. In addition, the continuous mining of underground and open pit mines may affect the vegetation within the mining scope and even reshape the local geological topography. Hence, “multidate” land use data of mining areas are important for evaluating the land resource changes and the ecological status of mining areas.

The periods with the most significant land use/cover changes in mining areas include the initial period of mining activities and the period of resource exhaustion [30,31]. Land use/cover changes are mainly characterized by the conversion from industrial and mining land to construction land, forest land, or grassland. In other stages of mining area exploration, the changes in land use/cover are relatively slight. Time series-based remote sensing data monitoring is a conventional means to analyze land use changes [32,33], whereas the reserves of mineral resources and the development demand of the local economy also profoundly impact land use changes. In this sense, the combination of these two factors can better explore the driving force of land use changes in mining areas from a deeper perspective. For example, coal mining activities in the Raniganj region, India, have started in the 18th century, resulting in a series of problems such as significant changes in land use patterns and excessive groundwater mining [34]. The land use data over 26 years, obtained via satellite data, can be used to effectively monitor the changes in land use types, thereby providing basic data for solving environmental issues. In the mountains of northwest Spain, to quickly restore the environment of mining areas and to protect the surrounding farmland and protection areas, ecological planning based on the current land use status in the mining areas was performed, along with artificially accelerating the reconstruction of original land types [35]. Different driving factors for changes in land use types make it more complex to implement remote sensing monitoring of land type changes in mining areas. In this case, other types of data may be adopted, making it difficult to maintain data identity and thereby complicating data analysis. These are also difficult points in the analysis of land use changes in mining areas.

Extracting the ground features of mining areas via the remote sensing index is also a common method to analyze land use changes. The relevant indices of construction land (normalized construction index), bare land (bare soil vegetation index), water area (normalized differential moisture index), and green vegetation (normalized vegetation index) are all relatively advanced [36,37,38], and the extraction results can also be mutually verified with the land use classification results. Overall, research on remote sensing classification of mining land use/cover is relatively sophisticated, but the accuracy of land use classification results in mining areas in the monitoring process is greatly influenced by the knowledge structure of researchers. In addition, the classification of tailing ponds is also one of the important problems in remote sensing monitoring.

## 3. Remote Sensing Monitoring of Landscape Ecology in Mining Areas

The balance and stability of the ecological state is an important prerequisite for the sustainable development of mining areas, and an intact ecosystem is crucial for improving the environment of mining areas. Remote sensing allows the dynamic monitoring of vegetation, animals, and landscapes in mining areas and can therefore facilitate the acquisition of change information, thus providing basic data support for ecological assessment.

### 3.1. Biodiversity Monitoring

**Figure 1:**
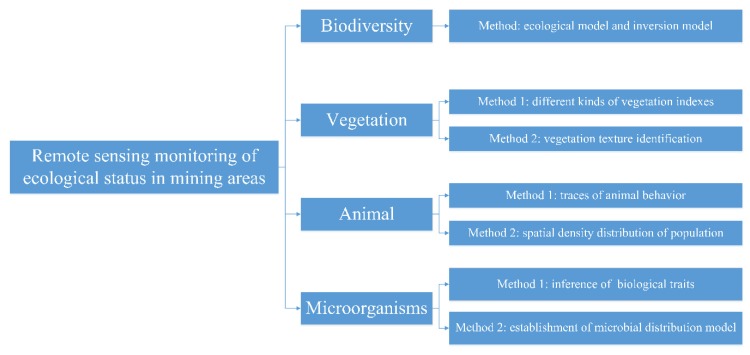
Biodiversity monitoring methods.

Due to the differences in physiographic conditions in mining areas, the biological species in different zones within the mine areas are also different. At present, research on the biodiversity of mining areas mainly focuses on vegetation, animals, and microorganisms. Mining areas contain a large variety of plant species [39] of numerous families and genera. The animal species in the mining areas are mostly soil-inhabiting animals with a small body size [40]. Although microorganisms are often partitioned into fungi, bacteria, and viruses, related studies mostly selected microbial communities as research objects [41]. A full understanding of the species and growth habits of plants, animals, and microorganisms in mining areas facilitates studies on biodiversity [42] and can also provide references for biodiversity monitoring via remote sensing in mining areas (Figure 1) [43,44,45,46]. Additionally, the environment of mining areas significantly influences plant and animal growth as well as the microbial community structure. Therefore, along with the remote sensing monitoring of the biodiversity and ecological status of mining areas, the monitoring results can be used to infer the changes of the surrounding environmental factors, which have great significance.

Vegetation type and quantity, as well as planting area and density, can be monitored with the use of remote sensing data. The vegetation index, as a common monitoring tool, has received considerable attention. At present, there are more than 200 vegetation indices published in the scientific literature, based on comprehensive remote sensing data [47,48]. Additionally, at least 27 models have been strictly subjected to biological verification for commercial promotion [49]. Numerous studies on the vegetation in mining areas have shown that the leaf area index (LAI) can identify vegetation types with different canopy structures [50], facilitating the rapid acquisition of vegetation types in mining areas. The degree of vegetation coverage in mining areas can be calculated via the NDVI, which can be used to judge the reconstruction status of the ecosystem [51]. At the same time, via object-oriented scale segmentation based on high-resolution images, we can easily obtain information of vegetation types in mining areas [52] and effectively estimate the regional vegetation coverage.

Compared with the studies on remote sensing monitoring of vegetation in mining areas, there are few studies on remote sensing monitoring of animals. On the bare land of mining areas, satellite remote sensing and unmanned aerial vehicle technology can be used to monitor animals [53,54]; however, in areas with dense vegetation, monitoring using remote sensing technology could be difficult due to leaf occlusion. Therefore, methods of animal behavior tracking and spatial density distribution could be used to deduce the animal distribution [44,45,55]. Under these circumstances, it is important to promote the use of free high-resolution satellite images for animal monitoring. Especially for those data with a spatial resolution up to 30 m or higher, animal activities and other related traces can be effectively identified on these images [56].

Unlike direct monitoring of animals and plants, commercial sensors cannot directly identify the physiological status of microorganisms. At present, research on the remote sensing monitoring of microorganisms involves two aspects: one is to infer the physiological status of microorganisms from other organisms, soil quality, and water quality, and the other is to establish the microbial distribution model [57,58,59]. It should be noted that the remote sensing monitoring of microorganisms and ecological communities is expensive and still in the experimental stage [57].

Although remote sensing monitoring of the species composition and the dynamic evolution of ecological communities is achievable in large mining areas or in areas with intensive mining exploration, a vast volume of data and documents is required in addition to the remote sensing data. In general, environmental monitoring in mining areas mainly focuses on vegetation monitoring, while the monitoring of animal and microbial communities in such areas needs to be strengthened.

### 3.2. Landscape Ecological Diversity

The landscape pattern of mining areas includes the types, quantities, spatial distribution, and configuration of ground features. For remote sensing data, the landscape patterns manifest as plaques of different land use types, and the randomness, uniformity, or aggregation of these plaques in space form the basis of the landscape pattern analysis of mining areas [60]. Currently, researchers commonly adopt landscape indicators such as plaque density (PD), Simpson diversity (SIDI), and contagion (CONT) to analyze and evaluate the landscape pattern based on the existing land use data of mining areas or land use results extracted by remote sensing classification [61]. For example, some scholars [62] have developed ecological restoration plans in the mining process by using the landscape index that is dominated by the plaque index and the landscape level index; considering the nearby reclamation project, other authors [63] have predicted the future landscape ecology of the mining areas according to the changes in the landscape pattern of the mining areas over 100 years and found that the landscape pattern of such areas resembled a natural landscape with high ecological stability.

Studying the landscape pattern of mining areas cannot fully meet the requirements of ecological restoration of such areas, and the specific features of the study area and the study objects need to be considered. To plan and design mining cities [64], a new landscape audit index is introduced and combined with the landscape pattern index for correlation analysis, thus making up for the defects of the analysis by the landscape pattern index alone. For the mining areas with landscape memory loss [65], the related animals are introduced to assist in the rebuilding of the new landscape, improve the ecological environment, and increase the species diversity. Some scholars [66,67] have integrated various land use types to establish a comprehensive landscape pattern and have simulated the landscape evolution to predict the soil erosion degree, the topography, and the landform, thus promoting the sustainable development of the mining industry and of other industries. Both the study of landscape patterns based on land use data and the prediction of the development of the study area based on landscape patterns have important reference significance for studying the landscape ecology of mining areas and restoring the damaged landscape pattern. Some results of previous studies lend themselves for the monitoring and restoration of the ecological environment of mining areas.

## 4. Remote Sensing Monitoring of the Environment in Mining Areas

Remote sensing allows the rapid acquisition of ground information, facilitating the timely monitoring of changes in vegetation, soil, atmosphere, and other environmental indicators when the climatic conditions and the topographical status of the mining areas change. Hence, remote sensing has become an important guarantee for improving the efficiency of mining area monitoring.

### 4.1. Extraction of Contaminated Vegetation

**Figure 2:**
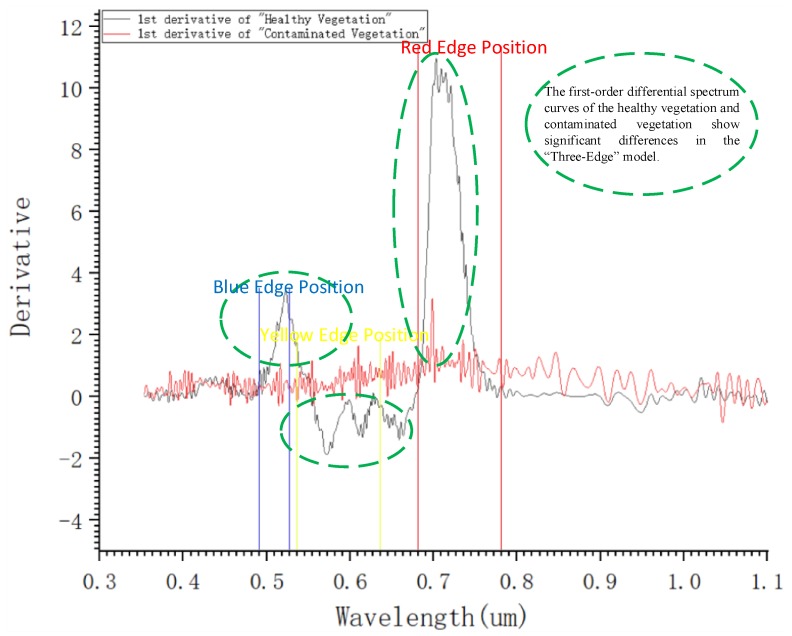
First-order differential spectrum curves of healthy and impacted vegetation based on the “Three-Edge” model (note: original spectrum data used to draw the figure are derived from the Jet Propulsion Laboratory, USA [72,73]).

Affected by mining activities, the growth status of contaminated vegetation can provide characteristic information based on differences in spectrum and texture. The methods of acquiring information such as the vegetation health status and the restoration effect from remote sensing images have been studied deeply, and impeccable monitoring systems have been widely applied [68]. Since the vegetation is generally subjected to different stress factors, its spectral characteristics are obviously different from those of naturally growing vegetation [47]. For example, via methods based on vegetation spectral features, such as differential spectrum estimation, vegetation index estimation, and “Three-Edge” model estimation (Figure 2), the biomass spectrum curve of the contaminated vegetation is generally lower than the normal value, while the influences of human factors on the restoration process may make the spectrum curves of some indices higher or even considerably higher than the normal values [69,70,71]. These spectral differences can monitor and analyze the vegetation growth dynamics and restoration effects, thus providing a theoretical basis for vegetation restoration. Most studies on the extraction of contaminated vegetation have focused on the extraction of chlorophyll biomass information, thereby directly reflecting vegetation health [71].

Currently, the differences between different vegetation types in mining areas, reflecting vegetation anomaly, can be distinguished through the vegetation index and biomass information, such as chlorophyll data [74,75,76]. However, the vegetation anomaly information obtained by remote sensing technology is not complete compared with that obtained by traditional chemical methods [69]. Hereinto, there is a certain relationship between the extraction effect of vegetation biomass information and the inversion model [77,78]. Currently, the commonly used inversion models include regression equation models, statistical and physical models, support vector machines, neural networks, and other empirical models [79,80,81]. Among these inversion models, regression equation models are widely used in studies on the correlation between measured vegetation biomass data and the vegetation index [82]. For example, some researchers [83] have used the regression model, the improved back propagation (BP) neural network model, and the support vector machine technique to invert the biomass of broadleaved forest, coniferous forest, shrub, and grassland in mining areas and compared the inversion results from different models. Based on their findings, the regression model was most suitable for inverting the biomass of broadleaved forest and coniferous forest, while the support vector machine technique was more accurate for the shrub and grassland areas with smaller plants. In addition, principal components analysis and the random forest model are also widely used in the inversion of vegetation biomass [84,85]. In general, most inversion models need to estimate and verify vegetation parameters via the use of field or laboratory data [86].

Considering that mining areas have a complex environment, it is necessary to study the dynamic changes of the impacted vegetation during growth. In recent years, some researchers have begun to consider the relationship between “geophysical variables” and the variation of vegetation physiological characteristics [87,88,89]. Among the related studies, the differences between the spectra of contaminated vegetation and healthy vegetation and the anomaly of biomass, such as chlorophyll of the contaminated vegetation, are important contents of current studies.

### 4.2. Soil Quality Monitoring

Soil pollution in mining areas is mainly a result of heavy metals (such as mercury, cadmium, copper, etc.), radioactive materials (such as uranium, thorium, radium, etc.), acidified substances, and salinized substances [90,91], which can all be effectively monitored by remote sensing. Among these pollutants, heavy metals are the most important ones [92,93], and their contents in soil can be obtained by the reflection spectrum or its deformation or be inferred from the physical and chemical soil properties (such as organic matter content, mechanical composition, nutrient content, pH, electrical conductivity, etc.) and vegetation growth [94].

Heavy metals in soil are generally monitored via remote sensing by establishing an inversion model of the heavy metal content based on the soil spectral reflectivity data measured in the laboratory [95]. When studying the contents and spatial distribution of heavy metals in soil, some authors [96,97,98], according to the size of the study area and other factors, have selected dozens or even hundreds of sample points for spectral correction to obtain benchmark data and predicted the large-scale heavy metal content on the basis of these sample points. As opposed to the direct monitoring of heavy metals, measuring the indirect inference of the heavy metal content on physical and chemical soil properties requires that the substance closely related to the heavy metal content are identified. For example, when studying the As content of a soil, the prediction results depend on the close relationship between the content of As and the levels of soil organic matter and iron or aluminum oxides [98]. Regarding the measurement of soil Zn levels, organic matter and clay minerals have a strong adsorption effect on Zn [96]; on this basis, the content of Zn in soil could be determined. In terms of vegetation growth, some studies [99] have shown that the contents of Pb, Cr, Cu, Ni, Zn, Cd, Hg, and As in soil are closely related to the biomass content of rice. Therefore, rice leaves can be used in studies on heavy metals in soil, allowing to directly determine the contents of related metals in soil. According to another study, hyperspectroscopy allows one to identify pollutants in “special” soil-tailings in tailing ponds [100] and to monitor the activity and flocculation of tailings [101,102]. In addition, more than 40 scholars [103] have jointly studied the global soil spectra in the visible-to-near-infrared spectrum scope and coded the spectral reflectivity or emissivity characteristics of the minerals, organic matters, water, and other components in soil into the database. This global soil spectral database serves as a reference for identifying soil pollutants in mining areas.

In addition to the above methods of obtaining the contents of heavy metals in soil, the geological index can reduce the spectral changes caused by topography through the band ratio and can highlight some metallic materials in soil [104]. Currently, the commonly used geological indices include the clay mineral index [105], the nonferrous mineral index, and the iron oxide index [104]. These remote sensing indices provide a basis for the rapid and qualitative monitoring of soil metal levels and other pollutants in mining areas [106,107]. The rapid monitoring of the relative pollutant content via the remote sensing index can supplement the quantitative inversion of the degree of soil pollution in mining areas, increasing the feasibility of soil quality monitoring in mining areas.

### 4.3. Water Pollution and Air Quality Monitoring

Rainwater erosion and surface runoff in mining areas lead to pollutant diffusion and, consequently, to groundwater pollution. The risks and paths of pollutant diffusion caused by surface runoff can be monitored and simulated through image data [108,109,110], thus providing a basis for the prevention and governing of water pollution. Conventional monitoring of water pollution in mining areas usually includes band combination transformation calculation by convenient empirical models to extract suspended substances in water or inversion of the water pollution status by combining the measured data of water sample points (Table 2). By applying the short-wave infrared imagery (SWIR) empirical model and the pollution concentration index, the spatial distribution of heavily polluted waters in the mining areas can be quickly understood [111,112]; by fitting the image spectral values with the measured data of water quality, information on water pollution can be accurately inverted [113,114,115]. These two water pollution monitoring methods can complement each other to meet the requirements of different research stages and purposes [111].

Similarly, particles in the atmosphere can cause floating pollutants to spread around. Especially in non-metallic mine areas, the contents of toxic particles in the atmosphere are often high, which can have even more serious impacts on the environment than water pollution. For example, when studying the spatial and temporal distribution of mercury atoms in the atmosphere of the mining areas based on LiDAR data, the space near the mining area should also be included in the research scope [116,117]. In recent years, low-cost small unmanned aerial vehicles, equipped with air analyzers and aerosol optical sensors, have been used to monitor open mining areas [118,119,120]. Such air analyzers can monitor CO, SO_2_, O_3_, and other gases, while the remote sensing data results can accurately fit PM_2.5_, PM_10_, and dust concentrations.

Water fluidity, water permeability, and gas diffusivity impede environmental monitoring in mining areas. However, with the further development of respective technologies, these difficulties will be solved, resulting in a higher accuracy of monitoring data.

## 5. Conclusions and Prospects

By combining the related research progress of remote sensing monitoring of the ecological environment in mining areas, this paper reviewed the status quo of the current technology from the three aspects of ground feature type monitoring, ecological status monitoring, and environmental status monitoring. The identification of ground feature types in mining areas is the basis of remote sensing monitoring, and the accurate identification of mining area boundaries provides the basic data for monitoring landscape ecology and the environment. Among the different monitoring methods used for mining area boundaries, the fuzzy clustering processing of land use status data can rapidly, easily, and accurately extract mining area boundary information. In remote sensing monitoring of mining areas, research on the diversity of the ecological landscape is relatively sophisticated, while the monitoring of animals and microorganisms is still in its infancy; in particular, the relatively high costs largely limit the monitoring of microorganisms. Remote sensing monitoring in mining areas mainly focuses on impacted vegetation and soil; although the research results are abundant, relatively few studies have considered water and air pollution. When monitoring the biodiversity of mining areas, vegetation monitoring mainly involves the identification and comparison of different plant species, while research on impacted vegetation in mining areas mainly focuses on the internal comparison of vegetation species. Land use/cover changes in mining areas are closely related to the ecological landscape diversity. They are interrelated with each other, but relatively independent. Generally, studies on the remote sensing monitoring of the ecological environment in mining areas are mostly isolated studies on a certain aspect, and there are few relatively comprehensive studies.

According to different global division criteria, there are numerous different types of mining areas, and the environmental impacts of mining are broad. The effectiveness of remote sensing technologies on monitoring in mining areas varies according to the differences in region, the mining method, and the mined commodity. For example, the soils of most mining areas contain iron, and as a result, the remote sensing monitoring technology on iron elements in mining areas is more mature than the technologies used for other mineral elements [121]. Due to their unique features, radioactive mineral resources are more recognizable in remote sensing images than non-radioactive mineral resources [122]. Compared with regions densely covered by vegetation and with underground mining areas, environmental monitoring via remote sensing is less complex in regions with bare vegetation [75,79]. In addition, differences of vegetation density in mineral mining areas also result in differences of monitoring effects when using remote sensing [79,87].

Our paper is, however, limited in several aspects. For example, we reviewed the remote sensing technology for monitoring the ecological environment in mining areas according to the monitoring objectives instead of the technology type. This was mainly done to emphasize the progress of this technique in various fields rather than to focus on the remote sensing technology itself.

Due to the limitations of remote sensing data, biodiversity monitoring in mining areas via remote sensing is not yet fully possible. Even for an advanced vegetation monitoring technique, there are few studies on the remote sensing monitoring of internal energy flows. However, in the future, with the further development of remote sensing satellites, remote sensing monitoring in mining areas will become more convenient, affordable, and faster, with an improved resolution and accuracy. At the same time, the stereoscopic monitoring by different remote sensing satellites and the comprehensive monitoring of the mining area environment with multisource data will be possible.

## Figures and Tables

**Table 1:** Remote sensing identification of mining area boundaries.

Identification Method	Data Types	Advantages	Disadvantages
Fuzzy clustering method: generating clusters similar to ground feature pixels [22,23]	Landsat land use data, Google Earth data	Can accurately and conveniently determine the extension scope of mining areas	Scope of mining areas should be relatively continuous and concentrated
Multivariate normal distribution: mining area data reflectivity, data set fitting [24]	Landsat-5 TM, Landsat-7 ETM, Landsat-8 OLI	Low time cost and economic cost	Authenticity of the results needs further verification
Vegetation index differential method: NDVI data difference based on time series [25]	Landsat-2 MSS, Landsat-5 TM, World View data	Can effectively identify changes in mining land	Precision of mining land boundary is uncertain, and the method has certain requirements regarding the surrounding environment
Snake model: identifying the mining area scope by curve evolution of image information [26]	Land observing satellite data: panchromatic data of 2.5 m and multispectral data of 10 m	High accuracy, high degree of automation, desirable processing ability for vast data volume	Model is difficult to be implemented and requires a higher data quality
Microwave interference method: adopting the binary-channel D-InSAR scheme of time series [27]	ALOS PALSAR data, high-resolution DEM data	Can continuously observe the fluctuation of boundary geodetic patterns	Observation accuracy depends on data accuracy and surface deformation

Notes: ALOS is Advanced Land Observation Satellite; PALSAR is Phase Array Type L-band Synthetic Aperture Radar.

**Table 2:** Remote sensing monitoring of water pollution information in mining areas.

Model Category	Monitoring Methods	Monitoring Content	Advantages and Disadvantages	Typical Cases
Empirical models	SWIR empirical model	Water bodies containing soil, water bodies polluted by coal dust	The required information can be obtained rapidly and conveniently; however, the qualitative analysis results are inaccurate and only suitable for heavily polluted areas.	Mining areas in the Tapajós River basin [111]
	Pollution concentration index			Dexing Copper Mine [112]
Analytic or semi-analytic model	Comprehensive nutritional index method	Water chlorophyll concentration, suspended matter content (algae, etc.), organic matter content, heavy metal content, etc.	More comprehensive water pollution information can be acquired, and accurate quantitative analysis can be carried out; however, a vast volume of measured data is required, and the operation calculation is complex.	Huainan mining area [113]
	Genetic programming			Albufera, Valencia [114]
	Multivariant stepwise regression model			Wansheng mining area [115]
